# The CD83 Molecule – An Important Immune Checkpoint

**DOI:** 10.3389/fimmu.2020.00721

**Published:** 2020-04-17

**Authors:** Linda Grosche, Ilka Knippertz, Christina König, Dmytro Royzman, Andreas B. Wild, Elisabeth Zinser, Heinrich Sticht, Yves A. Muller, Alexander Steinkasserer, Matthias Lechmann

**Affiliations:** ^1^Department of Immune Modulation, Universitätsklinikum Erlangen, Friedrich-Alexander-Universität Erlangen-Nürnberg, Erlangen, Germany; ^2^Division of Bioinformatics, Institute of Biochemistry, Friedrich-Alexander-Universität Erlangen-Nürnberg, Erlangen, Germany; ^3^Division of Biotechnology, Department of Biology, Friedrich-Alexander-Universität Erlangen-Nürnberg, Erlangen, Germany

**Keywords:** CD83, immune tolerance, autoimmunity, viral escape mechanism, Treg cells

## Abstract

The CD83 molecule has been identified to be expressed on numerous activated immune cells, including B and T lymphocytes, monocytes, dendritic cells, microglia, and neutrophils. Both isoforms of CD83, the membrane-bound as well as its soluble form are topic of intensive research investigations. Several studies revealed that CD83 is not a typical co-stimulatory molecule, but rather plays a critical role in controlling and resolving immune responses. Moreover, CD83 is an essential factor during the differentiation of T and B lymphocytes, and the development and maintenance of tolerance. The identification of its interaction partners as well as signaling pathways have been an enigma for the last decades. Here, we report the latest data on the expression, structure, and the signaling partners of CD83. In addition, we review the regulatory functions of CD83, including its striking modulatory potential to maintain the balance between tolerance versus inflammation during homeostasis or pathologies. These immunomodulatory properties of CD83 emphasize its exceptional therapeutic potential, which has been documented in specific preclinical disease models.

## CD83 – Gene Structure and Promotor Characterization

Since its discovery in 1992 (1, 2), CD83 has been extensively studied and been now treated as a promising potential therapeutic target. A recent review provided a short overview of the CD83 biology with a major focus on therapeutic applications using anti-CD83 antibodies and recombinant soluble CD83 (3). Here, we summarize in a deeper view the structure and control of the CD83 promotor, the newest analysis of the protein structure, and the regulatory functions of CD83 in immune response and tolerance.

Both, murine (muCD83) and human CD83 (hCD83) are composed of an extracellular V-type Ig-like domain, a transmembrane domain, and a cytoplasmic tail. The murine C*d83* gene is located on mouse chromosome 13 band A5, spans ∼19 kb and is composed of five exons and four introns (4). In particular, exon 1 encodes the 5′UT sequence, the translation initiation codon and the first 12 amino acids of the signal peptide. Exon 2 codes for the remainder of the signal peptide as well as 32 amino acids of the Ig-like domain. Exon 3 comprises the residual 65 amino acids of the Ig-like domain. Exon 4 contains the putative transmembrane region, and exon 5 encodes the 39-amino acid cytoplasmic tail and the large 3′UT sequence (5).

On the other hand, the human *CD83* gene maps to chromosome 6p23 (5) and both, the mu*Cd83* and h*CD83*, share the identically positioned translation initiation sequence (4). Although the mu*Cd83* gene structure has been well characterized in the past, the *CD83* promoter region has only been decoded in humans, i.e., human monocyte-derived dendritic cells (DCs). Here, a 261 bp-spanning minimal promoter (MP) region upstream of the translation initiation site was identified to drive hCD83 expression (6). Interestingly, this MP region lacks any maturation- and cell-type specificity. Additional studies in human DCs revealed a highly transcriptionally active module within the h*CD83* gene locus. This module was shown to consist of an upstream regulatory element (URE) of 164 bp, located 85 bp upstream of the minimal promoter (261 bp, MP-261), and a downstream enhancer (185 bp) within intron 2 of the CD83 gene. Here, the URE and the enhancer were reported to work synergistically *in trans* (7). Transcriptional activation is mediated by a complex framework of three interferon regulatory factors (IRFs) and five NFκB-transcription factor binding sites (TFBSs) involved in the exact arrangement of this tripartite structure in DCs, with NFκB-family members p50, p65, and cRel synergizing with IRFs including IRF-1, IRF-2, and IRF-5. Noteworthy, although CD83 is not exclusively expressed by mature DCs, but also by activated lymphocytes, this tripartite promoter complex is neither active in T- or B cell lines nor in primary activated T- and B cells (7).

In addition to this, a very recent study described the aryl hydrocarbon receptor (AhR) to be involved in the transcriptional regulation of the CD83 molecule (8). Bioinformatics analyses revealed two potential AhR-binding motifs (XRE) within the URE and the MP-261 of the human CD83 promoter. Following activation of AhR by the flavonoid quercetin, AhR was demonstrated to directly bind to the P-510 in human DCs, accompanied by a strong downregulation of CD83 mRNA and protein expression. Regarding the mode of action the authors hypothesize that the negative control of CD83 transcription by AhR might be either due to the association of AhR with NF_*K*_B, thereby modulating its activity, or due to a steric hindrance of adjacent AhR affecting the binding of NF_*K*_B to its site (8).

Thus, transcriptional regulation of the human CD83 molecule is not only controlled by “classical” immune cell-related transcription factors like NF_*K*_B and IRF, but also by environmental sensors like AhR.

## Structural Features of the CD83 Protein

**FIGURE 1:**
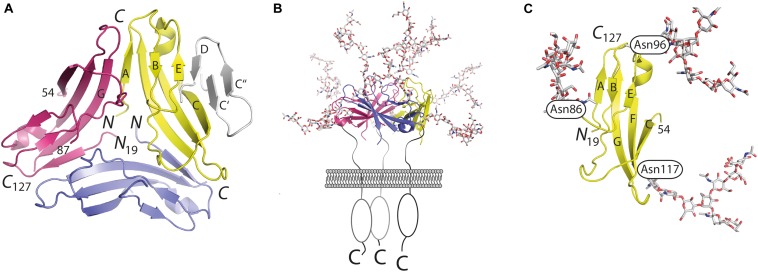
Atomic structure of CD83. Model for the presentation of trimeric full-length CD83 on cell surfaces in comparison to the monomeric structure of shedded soluble CD83. **(A)** Trimeric assembly as observed in the crystal structure of hCD83. The β-strands C′, C″, and D are not visible in the crystal structure of hCD83. For illustration purpose, they are added in gray to one monomer. **(B)** Presentation of trimeric hCD83 on cell surfaces and **(C)** glycosylated monomer formed by the soluble shedded variant of CD83. The structure illustrations have been drawn with program PyMOL (The PyMOL Molecular Graphic System. WL DeLano – Schrödinger, LLC, New York, 2010).

Orthologs of CD83 have been detected in more than 50 vertebrates including fish, reptiles, birds, and mammals. Between distant orthologs (e.g., fish and human), the sequence identity can drop to 25–28%. Exclusively one experimentally determined structure is available of these orthologs, namely hCD83 (9). Structural insight was gained by X-ray crystallography from three different crystal forms, in all of which hCD83 adopts a highly similar tertiary structure and homotrimeric quaternary assembly (Figure 1A). CD83 was crystallized using a truncated version of hCD83 that corresponded to residues 20–131 of the 205 residue-long gene product (UNIPROT ID: Q01151) (10). This crystallized fragment only encompassed the extracellular domain of hCD83 as compared to the bioinformatically inferred domain structure of full-length hCD83 (residues 1–19: signal peptide; 20–144: extracellular domain; 145–166: transmembrane region and 167–205: cytoplasmic tail).

The resolution of the crystal structure of hCD83 provided answers to issues discussed controversially in the past. The structural insights demonstrated that the extracellular domain of CD83 indeed folds into a V-set Ig domain. Human CD83 contains five cysteine residues in the extracellular domain that form two pairs of disulfide bonds. It is hypothesized that the fifth cysteine is hindered from forming an intermolecular disulfide bond by the presence of several protein surface-attached oligosaccharides (11, 12).

The interactions between the monomers are characterized by extensive homotopic contacts that bury as much as 1080 Å^2^ of the surface of each monomer within the trimer interface. Thus, the crystal structure suggests that hCD83 is assembled into a homotrimer when presented as a full-length integral membrane protein on the surface of, e.g., DCs. In contrast, the additionally observed soluble form of hCD83 is hypothesized to act as a monomer in solution (Figures 1B,C).

**FIGURE 2:**
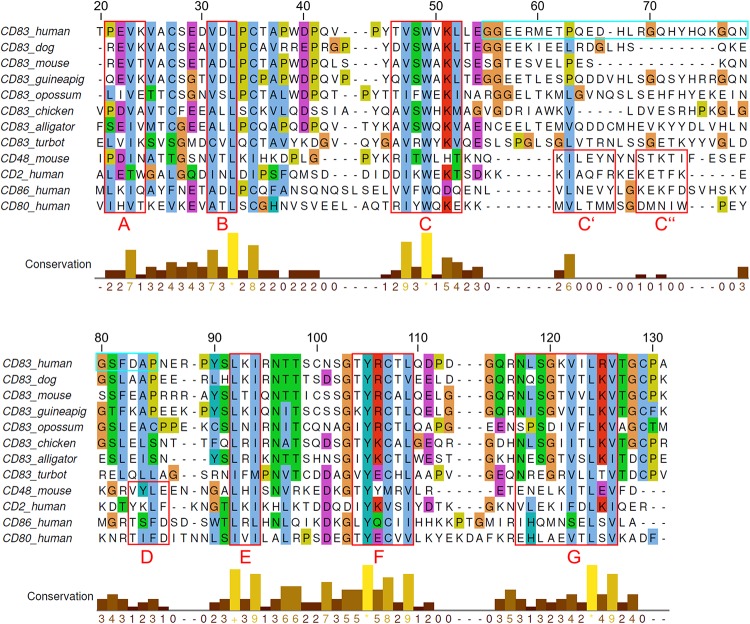
Multiple sequence alignment of CD83 orthologs and paralogs. The alignment includes eight CD83 orthologs from different species as well as four paralogous Ig-domains of high structural similarity. The sequence numbering of hCD83 is given on top of the alignment. Characteristic β-strands of Ig-domains are indicated by red boxes and labeled. A cyan box marks the sequence stretch that is not visible in the hCD83 crystal structure. Sequences were retrieved from UniProt and NCBI and the alignment was performed using ClustalOmega. The alignment of the paralogs was further adjusted using the structural information from PDB entries 5mixA, 2ptvA, 1qa9A, 1ncnA, 1i8lA. Visualization of the alignment was done with Jalview using the “Clustal” coloring scheme for residue conservation.

A sequence alignment of several representative CD83 orthologs and paralogs is shown in Figure 2. With respect to sequence conservation, the alignment reveals the presence of three distinct segments (Figure 2). The N- and C-terminal segments, corresponding to residues 20–54 and 89–129 of hCD83, respectively, displayed a significant sequence conservation. These segments contain the β-strands A, B, C, E, F, and G of the Ig-fold. Cysteines at position 35 and 107 form the canonical disulfide bridge, which is present in the vast majority of Ig-domains. The three remaining cysteines (Cys27, Cys100, and Cys129), present in hCD83, are moderately conserved among the CD83 orthologs, but not among the paralogs (Figure 2). Site-directed mutagenesis has revealed that all these three cysteines can be replaced by serines without losing the activity of soluble CD83 (9).

In contrast to the highly conserved N- and C-terminal segments, the middle segment is only poorly conserved among CD83 orthologs with respect to length and sequence (Figure 2). It is also noteworthy that all CD83 paralogs shown in Figure 2 form heteromeric interactions with other Ig-domains, which play an important role in signal transduction (9).

## Cd83 Expression

As mentioned above, CD83 protein is conserved among highly distinct species ranging from fish to mammals. While murine and human CD83 share 63% amino acid identity (4, 13), chicken and human CD83, and chicken and murine CD83 share 39 and 40% identity, respectively (14). Two protein isoforms of CD83 have been described in mice and humans: a membrane-bound form (mbCD83) (15) and a soluble form (sCD83) (16). Upon transcription, *CD83* mRNA is exported from the nucleus to the cytoplasm by an uncommon mechanism, involving the cellular RNA-binding protein HuR, the eukaryotic initiation factor 5A (eIF-5A), and the nuclear export receptor CRM1 (17). Concerning this, recent data reported the shuttle phosphoprotein APRIL (ANP32B) to be involved in the HuR-mediated nucleocytoplasmic translocation of *CD83* mRNA by acting as an adaptor protein that links HuR and CRM1 (18, 19). Further studies identified an additional RNA binding protein, namely AUF1 (hnRNP D), to regulate translation of *CD83* mRNA (20). However, the precise mechanisms regulating CD83 post-transcriptional processing and transport toward cellular organelles require future investigations.

Although CD83 is still one of the most prominent surface markers for fully mature human and murine DCs, including Langerhans cells (1, 15, 21), its expression is widely distributed among different cell types. These include B cells (22), activated CD4^+^ T cells and Tregs (18, 23), granulocyte-precursor cells (24), myelocytes (25), neutrophils (26), murine thymus epithelial cells (27) various tumor cell types (e.g., Hodgkin’s lymphoma) (28) and Epstein-Barr Virus transformed lymphoblastoid cell lines (29). Moreover, one study showed CD83 to be expressed by various immune cell types *in vitro* and *in vivo*, using a CD83-EGFP reporter mouse (30). The group further described a high and a low CD83 promoter activity in mature and immature DCs, respectively. However, while immature DCs lack detectable CD83 cell surface expression levels, CD83 is restored in the Golgi complex and endocytic vesicles (31, 32). Thus, the internal storage of CD83 can be transported to the cell surface immediately upon maturation. Interestingly, in monocytes and macrophages CD83 activity was undetectable, while resident B cell populations of spleen and lymph nodes showed a strong CD83 promoter activity. In-depth analyses revealed that pro-B and early pre-B cells lack any EGFP expression, whereas late pre-B cells showed a significant upregulation of EGFP expression, with the majority of naïve immature B cells still being EGFP positive. In contrast, CD8^+^ T cells as well as CD4^+^ T cells, including Tregs, effector memory cells, and central memory cells, were demonstrated to express CD83 exclusively upon activation (23, 30), with stronger promoter activity in CD4^+^ compared to CD8^+^ T cells.

Collectively, the CD83 molecule is expressed by a great variety of cell types, but mainly by activated immune cells like DC, B- and T cells as well as by thymus epithelial – and tumor cells. In the following section of this review, we will now highlight the proposed functions of CD83 among the different cell types.

## The Role of CD83 as Master Regulator in the Development of Adaptive Immunity

### CD4^+^ T Cell Development in the Thymus

The generation and characterization of a complete CD83 knockout (CD83^–/–^) mouse by Fujimoto et al. in 2002 highlights the essential role of CD83 expression by the thymic microenvironment and antigen presenting cells (APCs) for the selective development and peripheral egress of CD4^+^ T cells *in vitro* and *in vivo* (33). When comparing the phenotype of these animals with wildtype (wt) littermates, a striking reduction in thymic (68% less) and peripheral (75-90% less) CD4^+^ T cells was found, without affecting the phenotype, distribution, and development of other thymocytes. Crossing of CD83^–/–^ mice with AND^+/+^ mice, which carry major histocompatibility complex class II (MHCII)-specific TCR transgenes and thereby induce a positive thymocyte selection into the CD4 lineage, further affirmed the above. In experiments using bone marrow cells of either CD83^–/–^AND^+/+^ mice or AND^+/+^ mice that were transferred into irradiated CD83^–/–^ and wt littermates, both groups equally developed in wt mice but not in CD83^–/–^ recipient mice. Since CD83-deficient bone marrow cells gave rise to normal numbers of peripheral CD4^+^ T cells only when transplanted into wt recipients, the lack of CD4^+^ T cells was caused by extrinsic effects by the thymic microenvironment rather than an intrinsic defect in the CD4^+^ T cell itself. In mice, CD83 is equally expressed on thymic epithelial cells (TECs) and dendritic cells. The group additionally proved that exclusively the transfer of wt TECs but not wt DCs into CD83^–/–^ mice could restore the thymic CD4^+^ T cell development (33). Supporting this, a recent report demonstrated that the transmembrane domain of CD83 antagonizes the ubiquitin ligase MARCH 8, which in turn stabilized MHCII on cortical TECs. This novel functional pathway was essential for a normal thymic positive CD4^+^ T cell selection (27). Consistent with this, the critical role of CD83 during CD4^+^ thymocyte development was also confirmed by using a mouse mutagenesis screen. In particular, CD83 mutant animals with a homozygous missense mutation in the last exon of CD83 displayed a comparable aberrant phenotype as CD83^–/–^ mice did. Notably, this phenotype could be rescued by transgenic expression of CD83 in the mutant background (34).

### Treg Differentiation and Stability

Although endogenous CD83 expression on CD4^+^ T cells is not essential for normal thymic CD4^+^ T cell development (35), several T cell subpopulations express CD83 after activation (30, 36). While several studies revealed a modulatory function of CD83 on APCs (see below), the function of CD83 on T cell populations remained a long-time enigma. Recent studies clearly showed that a rapid and strong expression of CD83 upon activation is unique feature for murine Treg cells (23, 35). It has been demonstrated that only murine CD4^+^CD25^+^Treg have the ability to highly induce CD83 expression on transcriptional level, when compared to CD4^+^CD25^–^T cells (35). Further immunofluorescence staining and flow cytometric analyses confirmed that murine CD83^+^CD4^+^ T cells showed a concurrent upregulation of Treg specific markers upon activation, such as CD25, CTLA-4, GITR, Helios or NRP-1 (23, 35).

These murine data are further supported by studies in the human system, which also showed a distinct CD83 expression profile comparing anti-CD3/CD28-stimulated human effector versus regulatory T cells (23, 37). Upon TCR-stimulation *in vitro*, human CD4^+^ T cells upregulate CD83 expression transiently with a maximum expression at day 2 followed by a strong decline after day 3 (37). By contrast, expanded human CD25^*hi*^CD45RA^+^ Tregs already reached their *CD83* mRNA expression maximum 3 h after stimulation (23). Strikingly, TCR-stimulation of human and murine CD4^+^ T cells together with transforming growth factor beta (TGFβ) not only resulted in the expected differentiation into CD4^+^CD25^+^Foxp3^+^ iTregs but also in a sustained and stable CD83 expression (37, 38). In addition, immunofluorescence microscopy revealed CD83 to co-localize with CD25 on those cells (37). Strong expression of the high affinity IL-2-receptor alpha-chain CD25 is essential for Treg cell survival, due to their inability to produce IL-2 for survival and proliferation. Whether CD83 possesses a similar role in the stabilization of CD25 on Tregs compared to the stabilization of MHCII on TECs (see chapter 4a), needs to be elucidated.

Remarkably, forced CD83 expression in murine activated CD4^+^ T cells already induced their differentiation into Foxp3 expressing Tregs. Consequently, these cells acquired immunomodulatory functions and efficiently suppressed effector T cell-mediated immune responses *in vitro* as well as *in vivo* (35). Moreover, CD4^+^Foxp3^+^ T cells derived from BAC-transgenic mice expressing murine and human CD83 simultaneously, displayed an enhanced activated phenotype accompanied by an increased suppressive capacity in comparison to wt cells (39). The essential function of cell intrinsic CD83 expression in Tregs has been reported by studies of tissue specific conditional knockout (cKO) mice, in which CD83 is exclusively ablated in Foxp3^+^ Tregs. Compared to wildtype mice, these cKO mice showed a reduced percentage of Foxp3^+^ Tregs and an increased pro-inflammatory phenotype, which became even more prominent in aged mice. Moreover, elevated levels of autoantibodies against nuclear antigens were detected in the sera of young mice compared to the respective wt controls (38). This indicates an imbalance of the immune tolerance in Treg-specific CD83-deficient mice. *In vitro*, the CD83-deficient Treg cells possessed unaffected suppressive capacities and could be expanded similarly to wildtype Tregs, but produced higher levels of proinflammatory cytokines. In addition, adoptive transfer of either CD83-deficient or wt Treg cells together with effector cells into RAG1^–/–^ mice equally prevented intestinal inflammation. However, cKO mice developed an aggravated pathology with impaired resolution of inflammation in an experimental autoimmune encephalomyelitis (EAE) model (40). Regarding the demonstrated induction of iTregs by CD83 expression in murine naïve T cells the study showed that *in vitro-*differentiation of naïve CD4^+^ T cells from Foxp3-specific cKO mice resulted in strongly reduced numbers of Foxp3^+^ iTregs. Interestingly, this intrinsic loss of CD83 expression in Foxp3^+^ iTregs lead to reduced expression levels of Treg-specific differentiation markers and induced expression of inflammatory cytokines (35, 38).

**FIGURE 3:**
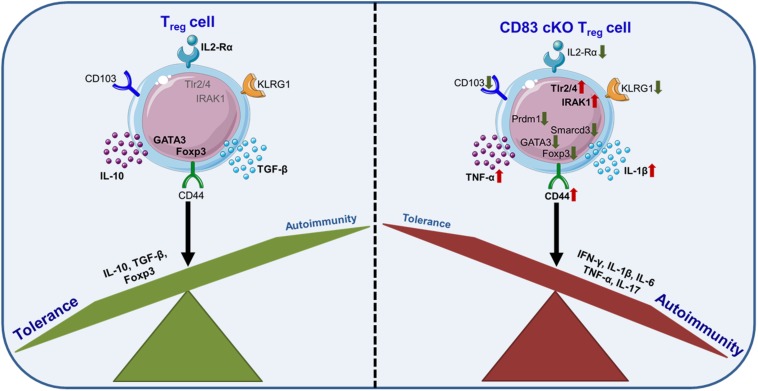
Endogenous CD83 expression is essential for Treg cell differentiation and stability. CD83-deficiency in Tregs results in decreased numbers of differentiated Tregs and less iTregs upon activation. Typical Treg differentiation markers, e.g., CD103, KLRG1 or IL-2Rα (CD25), are downregulated, while pro-inflammatory signaling pathways, e.g., IRAK1, and cytokines, e.g., TNF-α and IL-1β, are up-regulated. Overall, Treg-specific cKO mice showed an impaired tolerance.

These data were further supported by data derived from gene array analyses revealing a striking different gene expression profile of cKO Tregs compared to wt Tregs. Interestingly, while the lack of CD83 expression did not diminish the suppressive capacity of cKO Tregs, important Treg differentiation markers, e.g., CD25, KLRG1 and CD103, were downregulated (38). In particular, IL-2 signaling *via* CD25 plays an important role during Treg differentiation, expansion, and function (41). Thus, the diminished CD25 expression on CD83 deficient Tregs could already influence their stability. Further, KLRG1 is a late differentiation marker on T cells and the development of terminally differentiated KLRG1^+^ Tregs also depends on IL-2 signaling (40). Likewise, the CD103 molecule, a ligand for E-cadherin, has also been described as a marker for murine “effector memory”-like Tregs especially in the intestinal mucosa (42). Notably, CD83 expression in Tregs is not essential for Foxp3 expression (38). In summary, Treg intrinsic CD83 deficiency not only affects the generation of iTregs but also results in a lack of terminally differentiated Treg populations in the periphery of cKO mice. How CD83 stabilizes Treg differentiation upon activation remains yet to be elucidated. One potentially involved pathway could be deduced from studies with sCD83 on monocytes, which demonstrated that sCD83 modulates TLR4/MD2 signaling by downregulation of IRAK-1, switching from inflammatory signals to a tolerogenic outcome (see chapter 6; 43). Apart from this, another study demonstrated that activated Tregs release increased levels of sCD83 (23). Whether sCD83 reacts on Tregs or whether mbCD83 employs similar interaction pathways, has to be shown. Strikingly, Tregs from cKO mice expressed elevated levels of IRAK1, the shown CD83 interaction partner MD2, TLR2 and TLR4 compared to wt Tregs. Additionally, those cells exhibited reduced protein levels of the transcription factor NFATc2, which has been proposed to cooperate with Smad3, accompanied by an increased Foxp3 expression, higher Treg numbers and a mitigated inflammatory response (43). Thus, CD83 is proposed to modulate Treg differentiation at least partially via modulation of IRAK-1 expression (Figure 3). Noteworthy, other important regulators of Treg differentiation, as e.g., Prdm1 (BLIMP-1), Smarcd3 and GATA3, are also decreased in cKO Tregs.

In summary, it is remarkable that CD83 expression is not only essential on TECs or other APCs for thymic T cell selection and T cell activation, but in addition endogenous CD83 expression in T cell populations is indispensable for Treg differentiation and the stability of Tregs upon activation.

### B Cell Longevity, Homeostasis, and Class Switching

Since its discovery, CD83 has also been defined as a marker for B cells. Except pro-B and early pre-B cells, almost all resident peripheral B cell populations exhibit a strong CD83 promoter activity (30). Several studies demonstrate that CD83 expression correlates with B cell activation and is up-regulated after engagement of the B cell receptor, TLRs, or CD40 (44–46). The effects of CD83 expression by B cells have already been discussed in a recent review article (3). In short, one group found unaltered B cell numbers and tissue distributions in complete CD83^–/–^ mice, but defects in B cell (and CD4^+^ T cell) longevity in adoptive transfer experiments (44). However, this study lacks further evaluation of the observed reduced B cell longevity, i.e., whether it was due to a B cell intrinsic CD83 deficiency or caused by the loss of CD83 in other cell types. Noteworthy, transferred splenocytes of complete CD83^–/–^ mice contain strongly reduced numbers of peripheral CD4^+^ T cells, which could also influence B cell longevity (see also chapter 4a).

More mechanistic insights into the role of B cell-expressed CD83 were published by another group, who found reduced numbers of splenic marginal zone B cells and B1a cells in B cell-specific CD83 cKO mice (47). Moreover, cKO B cells exhibit an impaired MHCII and CD86 upregulation. Thereby, CD83 affects the CD86 and MHC-II expression on B cells by negative regulation of MARCH-1, as also demonstrated for DCs (48). Additionally, CD83 deficient B cells show enhanced proliferation and secreted more IL-10 upon activation with CpG but not LPS. Strikingly, germinal centers, which were formed after immunization of CD83 B cKO mice, contained elevated proportions of dark zone B cells. Surprisingly, this altered composition did not affect the affinity maturation of antibodies but ultimately led to increased IgE responses (47). In conclusion, these data revealed an essential modulatory function of CD83 in humoral immunity.

## CD83 and DC Activation

In spite of being described as a surface marker for mature DCs (mDCs), the precise biologic function of CD83 on these cells remains subject of controversial debate. In contrast to B cells, early studies of CD83^–/–^ bone marrow-derived dendritic cells (BMDCs) did not observe an influence of CD83-deletion on MHCII and CD86 expression (33). This view was later altered by a report of splenic DCs from CD83^–/–^ mice, which show reduced MHCII surface expression (49) and by another report of mice that carry a mutation within the TM-domain of CD83, which results in a lack of MHCII surface expression (48). Interestingly, the latter study provided evidence that also BMDC-expressed MHC-II and CD86 are negatively affected by CD83-depletion. Additionally, the authors demonstrated that the TM-domain of CD83 is both necessary and sufficient to stabilize MHCII and CD86 surface expression on BMDCs by antagonizing the actions of MARCH1 (48).

Given the central role of CD83 for stable surface display of MHCII and CD86, one would conclude that CD83 on DCs also critically influences the outcome of T cell stimulation (50–53). However, murine APCs which lack CD83 possess an equal stimulatory capacity compared to CD83 expressing cells (54).

Moreover, studies using DC-specific CD83 cKO mice demonstrated that this deletion rather increases/accelerates immune responses. Among others, it was shown that these CD83 cKO mice cleared bacterial infections more rapidly than their wt counterparts and DCs from those mice produced more IL-12 (55). In line with these findings, we demonstrated aggravated autoimmune responses in mice with CD83-deficient DCs, which *per se* display an over-activated phenotype and drive inflammatory T cell responses, presumably by interfering with Treg function (56). This enhanced stimulatory potential of CD83-deficient DCs both *in vitro* and *in vivo* is intriguing considering the reduced surface expression of MHCII and CD86 on these cells, which normally is a prerequisite for efficient T cell activation. Interestingly, excessive surface expression of MHCII, which is observed in DCs of MARCH1^–/–^ mice, is reported to perturb DC homeostasis. In this respect, MARCH1^–/–^ DCs show dramatically reduced IL-12 secretion and stimulatory potential toward CD4^+^ T cells (57, 58). Given its role as a negative regulator of MARCH1, CD83 might affect DC homeostasis by regulating MHCII expression. Since the expression of MARCH1 ceases upon DC maturation (59), the modulatory effect of CD83 might occur already in immature DCs (iDCs). Notably, CD83 and MHCII show only weak co-localization in human iDCs, which is in contrast to mDCs (31). Peptide-MHC-II complexes are ubiquitinated in early endosomes, which targets them for lysosomal degradation (60). Thus, the localization of CD83 in recycling endosomes of iDCs (31) might be a critical regulator of endosomal sorting of MHCII either back to the membrane or to multivesicular bodies for degradation. This sorting procedure assures efficient antigen presentation in mDCs versus iDCs (61). Interestingly, murine conventional DCs (cDCs) with defective MHCII ubiquitination are less capable of inducing antigen-specific CD4^+^ T cell proliferation (58), whereas CD83^–/–^ DCs exhibited increased potential to stimulate antigen-dependent T cell responses (49, 56).

Taken together, these findings suggest that CD83 impacts on antigen-presentation by modulating the endosomal sorting process in iDCs. Additionally, CD83-depletion – despite causing reduced MHCII and CD86 surface display – conveys an aberrantly activated phenotype to DCs leading to enhanced protective effect against bacterial pathogens as well as adverse autoimmune responses. This renders CD83 an important modulator of DC phenotype and function.

## CD83 Binding Partners and Signaling Cascades

Although the distribution of CD83 expression has been unequivocally unraveled using reporter animals (30) and extensive cell phenotyping (62), the receptor or ligand for CD83 (CD83L) remained elusive for a long time. Most studies that aimed to elucidate CD83 binding partners focused on the interaction of sCD83 with its putative receptor. It was demonstrated that murine B cells (63), human iDCs and mDCs (64) as well as activated CD8^+^ T cells (65) can bind sCD83, but none of these studies provided the definite report of a probable interaction partner. Interestingly, the formation of dodecameric sCD83 multimers was reported as a prerequisite for binding to activated human primary T cells and T cell leukemic cell lines, which is absent when using a conventional dimerized sCD83 molecule (65). Since binding studies with a dimeric sCD83 revealed that human monocytes as well as human DCs express a CD83L (64, 66), this suggests different avidities of sCD83 toward distinct ligands. However, there have been several reports describing CD83-interaction partners: first and as already stated above, the TM-domain of CD83 interacts with the E3-ubiquitin-ligases of the MARCH-family, resulting in stabilization of CD86 and MHC-II (27, 48). Second, in human mDCs CD83 is bound by GRASP55, an integral component of Golgi architecture and transport, which is crucial for efficient surface display of CD83 (67).

Regarding sCD83, a more recent study by Bates and colleagues incited an interesting new perspective on CD83 binding partners, suggesting that CD83 acts in a homotypic way (55). This notion is reinforced by the fact that many cell types, which have been reported to bind sCD83, indeed express mbCD83. For instance, binding of sCD83 to human T cells required their prior activation using agonistic anti-CD3/CD28 antibodies (65), a treatment that also induces mbCD83 expression (62). Additionally, the model of homotypic interaction integrates both the immune-regulatory effects of sCD83 and immunomodulatory function of mbCD83: if one interaction partner is missing, immune responses are more likely to derail. Interestingly, the cytoplasmic tail of CD83 lacks any consensus signaling motif and therefore the homotypic interaction of CD83 may act as a scaffold to facilitate the recruitment of additional proteins as signal transducers. This might also account for the different cellular distribution of CD83L when either using dimeric or multimeric sCD83 (65).

However, this self-interaction is not sufficient to explain the regulatory effects of sCD83 on monocytes (68), which – in contrast to DCs – do not express mbCD83 in the steady state (32). Thus, a recent study elucidated the impact of sCD83 on monocytes by demonstrating sCD83 binding to MD-2 and the TLR4 complex (69). The authors suggested that upon binding to MD-2, sCD83 initiates an anti-inflammatory TLR-signaling cascade leading to long-term depletion of IRAK-1, which causes unresponsiveness to further TLR stimulation. Interestingly, Tregs that are devoid of CD83 display elevated levels of IRAK-1 (see chapter 4b) (38), and a similar phenotype is observed in CD83-deficient DCs (56). Subsequently, both cell types exhibit an aberrant activation profile and are prone to propagate autoreactive immune responses in the absence of CD83.

A very recent study brought up a new view on the mode of action of sCD83 in the regulatory network of immune responses: by binding to CD154 on Th2 cells, sCD83 represses the apoptotic inhibitor Bcl2L12, thereby promoting cell death of this particular T cell subset (70). As a corollary, administration of sCD83 to animals with allergic rhinitis (AR) alleviated disease symptoms by restricting the Th2 response. Moreover, patients with AR showed significantly reduced sCD83 serum levels, which were inversely correlated with IgE-levels (70). Since B cells are presumably the major source of naturally occurring sCD83 (16, 71), these new data perfectly fit the Th2-skewed immunity observed in mice with CD83-deficient B cells (47).

Although mbCD83 may also interact with binding partners of sCD83, this has not yet been experimentally proven for the above-mentioned proteins. Therefore, the future elucidation of (putatively) further interaction partners on other cell types will unravel the entire complex network by which CD83 modulates and orchestrates immune responses. In summary, the notion that mbCD83 acts in trans to modulate ongoing immune responses in a similar way as sCD83 could explain the disturbed cellular and humoral immunity in CD83 cKO animals (38, 47, 55).

## CD83 in Health and Disease

Next, we discuss the current research examining the contribution of CD83 to the balance of the homeostatic and pathologic immune system *in vivo*.

### Bacteria

As mentioned above, the expression of CD83 on DCs is a critical determinant of the host immune response against bacteria. Mice with CD83-deficient DCs show improved resistance upon challenge with the extracellular enteropathogenic strain *Citrobacter rodentium* (55), and we further demonstrated enhanced clearance of intracellular bacteria like Salmonella or Listeria (56). In both settings, DCs from CD83 cKO mice react to bacterial challenge with increased expression of the cytokines IL-23 and IL-12. Interestingly, human DCs secrete considerable amounts of sCD83 when exposed to different commensal bacteria strains (72). These data suggest a crucial role of DC-expressed and -derived CD83 for the regulation of intestinal homeostasis and anti-bacterial immunity. While deletion of CD83 in DCs results in enhanced Th1 and Th17 T cell responses, B cell specific depletion of CD83 has quite different effects on the immune responses to pathogens. Upon infection with *Borrelia burgdorferi*, wt B cells display an enhanced pathogen clearance compared to CD83-deficient cells, which was associated with increased IgE serum levels suggesting a shift toward Th2 responses (47). Interestingly, earlier studies already reported that enforced expression of CD83 on B cells dramatically interferes with their ability to mount efficient antibody-responses against model-antigens as well as against infection with the parasite *Leishmania major* (45, 73). Thus, B cell-expressed CD83 is a critical regulator of humoral immune responses to pathogens, probably by disturbing the organization of germinal center reactions (47). Since B cells are a major source of sCD83 (16), compromised antibody production in CD83-overexpressing animals might also arise from a general dampening of the immune response. Furthermore, also human polymorphonuclear neutrophils acquire CD83 expression, but not MHCII or CD86, during acute bacterial infection. However, the biological relevance of this phenomenon remains to be elucidated (26). Moreover, in a model of neonatal exposure to Lipopolysaccharide (LPS), mice show a delayed onset and diminished severity of myelin oligodendrocyte glycoprotein (MOG)-induced EAE, compared with vehicle-exposed animals. Splenic CD11c^+^ cells from LPS-exposed animals exhibit reduced MHCII and CD83 expression during EAE. MOG-treated APCs from LPS-exposed mice stimulated less T lymphocyte proliferation but increased expansion of CD4^+^FoxP3^+^ T cells compared to APCs from PBS-exposed littermates. These findings support the concept of early life microbial exposure that influences the immune modulating capacity of APCs and neuroprotective regulatory T cells and that CD83 is part of the mechanism (74).

Collectively, these data reveal an important modulatory function of CD83 upon encountering pathogens, via sustaining the balance between tolerating commensals versus clearing harmful bacteria.

### Viral Infections

**FIGURE 4:**
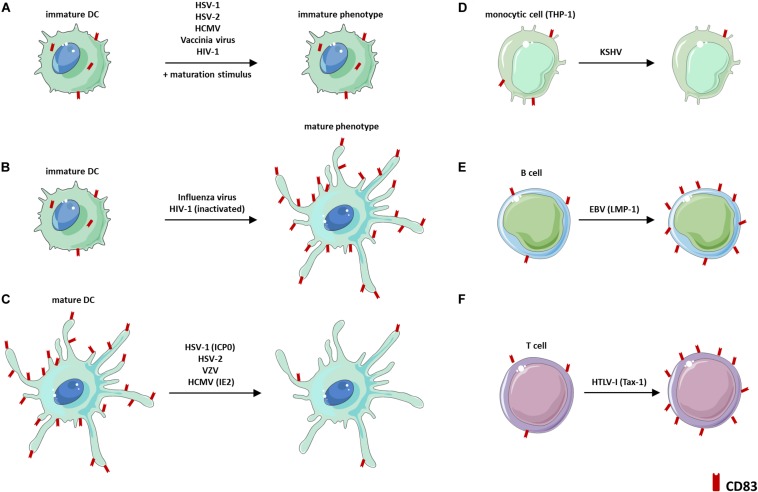
Virus-induced modulations of CD83 expression. **(A)** Viruses that block DC maturation and thus CD83 expression. **(B)** Viruses that induce CD83 expression *via* activation of a mature DC phenotype. **(C)** Viruses that mediate a downregulation of CD83 expression on mature DCs. **(D)** Reduction of CD83 surface expression during latent KSHV infection of THP-1 cells. **(E)** Induction of CD83 expression on B cells *via* EBV-encoded LMP-1. **(F)** Induction of CD83 expression *via* HTLV-1-encoded Tax-1. DC: dendritic cell, EBV: Epstein-Barr Virus, HIV-1: Human immunodeficiency virus-1, HCMV: Human cytomegalovirus, HSV-1: Herpes simplex virus type-1, HSV-2: Herpes simplex virus type-2, HTLV-1: Human T-cell leukemia virus type-I, ICP0: Infected cell protein 0, IE2: Immediate early 2, KSHV: Kaposi’s Sarcoma-Associated Herpesvirus, VZV: Varicella-zoster virus.

Viruses are highly adapted to their hosts and possess sophisticated strategies to support their own replication. Evidence is accumulating that a plethora of distinct viruses not only directly modulate CD83 expression levels, but also indirectly via, e.g., hijacking DC maturation (Figure 4).

Especially herpesviruses, which are capable of establishing latency upon lytic primary infections, have acquired immune evasion mechanisms targeting CD83. One prototypic member among the α*-herpesviridae* is Herpes simplex virus type-1 (HSV-1). In this context, HSV-1 has been shown to inhibit cytokine-induced maturation of iDCs, including upregulation of CD83 (75) (Figure 4A). Additionally, HSV-1 targets CD83 for its degradation in infected mDCs leading to a strong reduction of intracellular as well as surface-exposed CD83 protein levels (77, 78; Figure 4C). Within the tripartite gene expression cascade of HSV-1, the immediate early gene product infected cell protein 0 (ICP0) was proven to be essential and sufficient to induce the proteasome-dependent, but ubiquitin-independent, CD83 degradation in mDCs (76). Besides the loss of CD83 protein expression on directly HSV-1-infected mDCs, it has been reported that uninfected bystander mDCs also display a severe reduction of CD83 (77). This bystander effect was attributed to so-called L-particles, which are released by infected cells upon an HSV-1 infection and are non-infectious themselves, due to the lack of the DNA-containing capsid. However, it was suggested that L-particles are transmitted to bystander cells most likely to shape the cellular micro-environment in benefit of the virus (78). Apart from that, levels of sCD83 were shown to be unaffected during HSV-1 infection of mDCs, which excludes CD83 shedding to account for the loss of CD83 surface expression (79). Yet, the precise molecular mechanism of HSV-1-mediated CD83 degradation remains elusive. Thus, it is not known so far, whether HSV-1-encoded ICP0 directly targets CD83 on infected as well as bystander mDCs or whether ICP0 induces distinct signaling pathways that lead to CD83 downmodulation. Noteworthy, also the α-herpesviruses HSV-2 and Varicella-zoster virus (VZV) hamper the expression of CD83 on DCs (Figures 4A,C). In particular, HSV-2 blocks DC maturation (80), and thus CD83 surface expression, and additionally induces CD83 degradation after infection of mDCs (81). Furthermore, VZV also selectively inhibits CD83 expression on mDCs upon infection. In this way, VZV efficiently spreads inside the host by hijacking these migrating immune cells (82, 83). Notably, also the β-herpesvirus human cytomegalovirus (HCMV) was found to block LPS-induced maturation of DCs (84) (Figure 4A) and to target CD83 for immunomodulation when infecting mDCs (86, 87; Figure 4C). Regarding this, Senechal et al. demonstrated a strong reduction of CD83 surface expression on HCMV-infected mDCs concomitant with an induction of sCD83 levels in the respective cell culture supernatants (85). Remarkably, the latter observation was implicated to impair the T cell-stimulatory capacity of mDCs, thus interfering with an effective antiviral immune response (85). In contrast, a recent publication revealed that sCD83 levels were unaffected upon HCMV infection of mDCs. This apparent discrepancy is likely based on the different HCMV strains that were used among these studies (86). However, Heilingloh et al. provided new insights into the mechanism of CD83 reduction on HCMV-infected mDCs (86). The authors showed that the HCMV-encoded major immediate early 2 (IE2) protein is sufficient to induce a proteasome-dependent CD83 degradation of both surface-displayed as well as intracellular CD83 protein. These results are reminiscent of the ICP0-dependent CD83 downmodulation in HSV-1-infected mDCs (76, 79). Besides the α- and β-herpesviruses described above, also the γ-herpesvirus Kaposi’s Sarcoma-Associated Herpesvirus (KSHV) impairs CD83 expression during latent infection of the monocytic cell line THP-1 (87) (Figure 4D). In contrast, the Epstein-Barr Virus (EBV)-encoded LMP-1 protein was found to promote the expression of CD83 on B-cells, in the absence of an infectious insult, dependent on NFκB (29) (Figure 4E).

Interestingly, modulation of CD83 expression, either directly or indirectly *via* affecting DC maturation, is not a unique feature to *herpesviridae*, but is also mediated by, e.g., the human immunodeficiency virus (HIV-1) or Human T-cell leukemia virus type-I (HTLV-I), which belong to the family of *retroviridae*. Regarding this, it was proven that the HIV-1-encoded protein Vpr induces transcriptional downmodulation of CD83 in macrophages as well as DCs, while the latter observation was additionally found to be present on both directly infected and bystander cells (88, 89). Moreover, Vpr hampers DC maturation, accompanied by an inhibited CD83 upregulation (Figure 4A) and an inefficient activation of antigen-specific T cells for viral clearance (89, 90). Contrasting the downmodulation of CD83 upon HIV-1 infection, the exposure of DCs to inactivated HIV-1 virions or recombinant gp120 (HIV-1 strains Ada or IIIB)/Tat protein results in the upregulation of CD83 expression levels due to the induction of a mature phenotype (91, 92) (Figure 4B). However, one group reported that gp120s from distinct HIV-1 strains impair DC maturation and CD83 expression (93). Apart from HIV-1, HTLV-I induces the expression of mbCD83 and sCD83 by T cells, via activation of NFκB by the viral-encoded protein Tax1 (94) (Figure 4F), which mirrors the effect of EBV-encoded LMP-1. Since enforced expression of CD83 in T cells is known to confer a regulatory phenotype (35), this mechanism might be employed by HTLV-I to subvert antiviral immunity. Furthermore, vaccinia virus and influenza A virus also indirectly interfere with CD83 expression on DCs *via* differentially manipulating their maturation phenotype (97, 98; Figures 4A,B). Particularly, while vaccinia virus inhibits the upregulation of CD83 expression during DC maturation (95), the acute influenza A virus fosters DC maturation accompanied by induction of CD83 expression and the efficient stimulation of cytotoxic effector T cells (96).

The multitude of distinct virus families, which have independently evolved strategies to directly or indirectly target CD83 expression, underscores the vital role of CD83 during the activation of immune responses and thus the involvement in controlling (persistent) viral infections.

### Autoimmunity

Autoimmune diseases represent a family of at least 80 illnesses that share a common pathogenesis: an immune-mediated attack against the body’s own organs. Treatment of autoimmune diseases was highly improved during the second half of the 20th century. However, these treatment options proved difficult due to the progression of autoimmune disease prior to clinical diagnosis. Thus, much of the current investigation aims to shift the research focus toward immunomodulation. Understanding the effects of specific immune modulating interventions can elucidate definitive molecular or cellular checkpoints of the complex inflammatory networks which modulate autoimmune diseases. Given the important role of CD83 for immune responses to non-self, it is not surprising that there are several reports of CD83 being involved in autoimmune processes (38, 39, 55, 97–110). A recent report summarized immune-modulating functions of sCD83 therapy in models of multiple sclerosis, autoimmune uveitis and systemic lupus erythematosus (3). Here, we focus and discuss the very promising data about CD83 related therapy in rheumatoid arthritis, inflammatory bowel diseases, and diabetes mellitus:

#### Rheumatoid Arthritis

**FIGURE 5:**
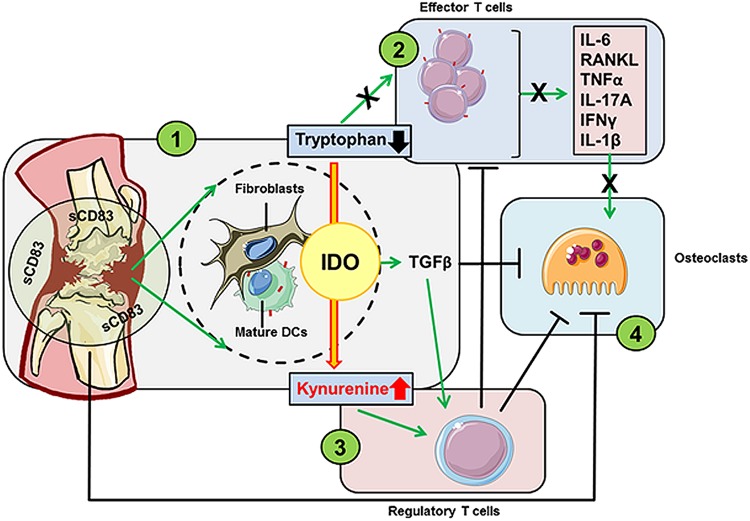
sCD83 has strong modulatory capacities in the murine AIA model for RA. sCD83 within the synovial cavities has four major striking effects on the pathogenesis of arthritis: (1) sCD83 induces both, the enzymatic and signaling activity of IDO in DCs as well as synovial fibroblasts. IDO leads to local tryptophan degradation and increased kynurenine levels, thereby (2) hampering the proliferation and differentiation of effector T helper cells and the subsequent reduction of proinflammatory cytokine levels. Furthermore, the increasing kynurenine to tryptophan ratio provides an anti-inflammatory environment and (3) promotes the differentiation of Tregs. This effect is further enhanced by TGFβ, a key cytokine of the non-canonical signaling pathway of IDO. The induction of TGFβ and Tregs, and the reduced levels of proinflammatory cytokines within the knee joints, (4) hamper osteoclast formation and activity. Moreover, sCD83 has a direct inhibitory effect on the osteoclastogenesis.

Rheumatoid arthritis (RA) is a chronic autoimmune disease that goes along with progressive articular damage, functional loss, and comorbidity (111, 112). Interestingly, elevated levels of sCD83 were detected in the synovial fluid of RA patients (105). It seems reasonable to assume that determination of sCD83 serum levels in patients with autoimmune disorders may serve as early prognostic biomarker of autoimmunity to predict and treat otherwise serious conditions. Importantly, this expression of sCD83 in early stage RA patients was unaffected by anti-TNF-α treatment (106). Moreover, CD83 (e.g., in B lymphocytes) possesses different regulatory functions of disease risk variants in RA (107, 108). Interference with autoimmune-mediated cytokine production is a poorly developed approach to treat autoimmune and inflammatory diseases, such as RA. A very recent study revealed that sCD83 enhances the resolution of autoimmune antigen-induced arthritis (AIA) by strongly reducing the expression levels of cytokines such as IL-17A, IFNγ, IL-6, and TNF-α within the knee joints. Noteworthy, also the expression of RANKL, osteoclast differentiation, and knee joint destruction was significantly inhibited by sCD83-treatment. Moreover, osteoclastogenesis experiments revealed an impaired osteoclast-specific phenotype in sCD83 treated cultures. These cells revealed a reduced fusion- and resorption capacity and showed a decreased expression of, e.g., *Oc-stamp*, *Mmp9*, *Trap*, and *Ctsk.* Blocking experiments, using anti-TGFβ antibodies further revealed that also TGFβ is mechanistically involved in the sCD83 induced reduction of bone destruction and cartilage damage as well as enhanced resolution of inflammation. Resolution of arthritis was associated with increased numbers of regulatory T cells within the synovium of sCD83-treated AIA-mice in an IDO-mediated manner (109). Treatment with sCD83 resulted in to long-term and antigen-specific modulation of the immune response in arthritis (summarized in Figure 5). Mechanistically sCD83 led to (i) upregulation of IDO and TGFβ, (ii) reduction of auto-aggressive Teff cells, (iii) induction of Treg cells and (iv) a direct impairment of osteoclastogenesis.

Taken together, treatment with sCD83 represents a promising approach for the resolution of autoimmune disorders, like RA, via downregulating cytokine production, and inducing regulatory T cells.

#### Inflammatory Bowel Diseases (IBD)

Pathologies of Crohn’s disease (CD), ulcerative colitis (UC) as well as non-infectious inflammations of the bowel originate from a dysfunctional immunological response against harmless microbial antigens in the gastrointestinal (GI) tract, which leads to a breakdown of immunological tolerance. Extensive research revealed that CD83 expression on different colonic leukocyte subpopulations, such as B cells, DCs, naive CD4^+^ and CD8^+^ T cells as well as Tregs plays an essential role in the context of intestinal immune homeostasis (23, 55, 100). In IBD patients, an accidental activation of DCs by microbial antigens leads to the induction of Th1 and Th17 cell immune responses. These responses are characterized by exaggerated release of pro-inflammatory cytokines with further activation of tissue macrophages and granulocytes, promoting inflammation in the GI tract (113). Different *in vitro* and *in vivo* experiments using DC-specific CD83 knockout animals demonstrated that mucosal DC activation and thus immune homeostasis is, in part, regulated by homotypic cell-cell interactions *via* surface-expressed CD83. On intracellular level, CD83 modifies the immune response through the mitogen-activated protein kinase pathway by inhibiting p38a phosphorylation and thereby DC activation (55). In addition, analyses of Foxp3-specific cKO mice in a transfer colitis model affirmed that expression of CD83 not only on DCs but also on Tregs plays an important role in intestinal immune regulation and homeostasis in IBD. In these experiments, the transfer of total CD4^+^ T cells from these cKO animals into Rag^–/–^ mice led to exaggerated colitis symptoms characterized by reduced survival, massive weight loss, and strong manifestation of clinical relevant parameters. Functional analyses further showed that this perturbed resolution of inflammation was caused by a diminished capacity of CD4^+^ T cells from cKO mice to terminally differentiate into effector Tregs upon activation (38). In a murine colitis model induced by dinitrobenzene sulfonic acid (DNBS), an outstanding high number of CD83^+^ leukocytes infiltrated the inflamed GI tract and even more interestingly, colonic cell populations were demonstrated to release sCD83 upon disease occurrence. Further investigations of the sCD83 molecule revealed its potential therapeutic capacity in the context of IBD. Strikingly, the application of sCD83 resulted in a remarkably reduced mortality in DNBS-treated mice. Weight kinetics supported the protective effect of sCD83 in DNBS-treated animals losing less weight and showing better recovery. Histological analyses of the colon suggested that the effect of sCD83 relies on decreased inflammatory leukocyte infiltration into the colonic tissue as well as ameliorated destruction of the colonic structure, and reduced loss of goblet cells (100). Additional experiments demonstrated that administration of sCD83 resulted in less expression of inflammatory cytokines such as TNF-α, IL-1β, and IL-6 in the colon.

Consistent with the findings in the EAE and RA models (3, 98, 109), IDO substantially contributed to the protective sCD83 effects in experimental colitis (100).

#### Diabetes Mellitus

Recently, Juhas et al. showed that decreased sCD83 plasma concentrations significantly correlated with disease progression in long-standing complication-free juvenile diabetic patients (110). Loss of sCD83 levels in the plasma of these patients coincided with increased HbA1c levels, a factor associated with disease progression. Interestingly, and similar to the observation in RA patients, sCD83 expression seems to be independent from TNF-α levels. Based on these findings, the authors proposed that sCD83 therapy might also have great potential in the context of long-term diabetes.

In summary, the beneficial effects of sCD83 described above, strongly suggest that this molecule could be widely applicable for the treatment of acute systemic inflammatory complications as well as chronic autoimmune inflammatory diseases. In addition, sCD83 has also been successfully used in preclinical transplantation models by us and others (114–117). However, clinical trials are essential and the next step in order to confirm the therapeutic efficacy of sCD83 in humans.

### Pregnancy

Maintaining the balance between immune tolerance and defense is also of significant relevance during pregnancy. As CD83 is a potent mediator in the control of immunity, it is not surprising that some studies reveal an association between CD83 expression as well as sCD83 upregulation and pregnancy. The control of the fetal-specific tolerance of the maternal immune system is quite complex and has not yet been completely understood. While more decidual DCs can be detected in women with recurrent miscarriage at 8 weeks’ gestation than in matched normal controls, there is no significant difference in the decidual CD83^+^ DC density (118). The authors also emphasize that it was unclear whether the CD83^+^ DC population in the decidua possess a stimulatory or inhibitory potential of a maternal anti-fetal immune response. So far, it is known that during pregnancy changes occur in DC, T, and B cell activities. It is proposed that immune tolerance is favored in the second trimester being gradually reversed in the third trimester (119). Various tolerogenic cell types in the maternal-fetal interface were suggested, such as, e.g., tolerogenic DCs, Tregs, and IL-10-producing B10 cells (119–121). As mentioned above, CD83 signaling can induce the differentiation of all of these tolerogenic cell types. Apart from this, IDO activity, also likely induced by CD83, has been shown to be essential for fetal tolerance (119, 122). In this respect, a striking recent murine study revealed that B and T lymphocytes, but not DCs, upregulate CD83 expression at day 14 of pregnancy (71). Moreover, the sexual hormone progesterone induces CD83 expression in murine T and B cells, but also in DCs *in vitro*. The authors further observed increased levels of the immunomodulatory sCD83 in advanced pregnancy and identified B lymphocytes as the major sCD83-producing cell type. In a very recent study, the same group also showed a correlation of poor pregnancy outcome and reduced serum levels of sCD83 using a CBA/JxDBA/2J mouse model of pro-inflammatory-mediated pregnancy disturbances. Regarding progesterone induced CD83, the group demonstrated that splenic B cells treated with progesterone decreased the expression of the metallopeptidase inhibitor 1 (TIMP1), mCD83 expression and sCD83 release, while TIMP1 treatment increased sCD83 levels *in vitro* (123).

Thus, deciphering the function of CD83 in fetal-specific tolerance and the mechanisms of sCD83 release by metalloproteinases and their inhibitory molecules will require further investigations.

## Concluding Remarks

During the last decades it has become clear that the CD83 molecule plays a very important role in the orchestration of proper immune responses and the subsequent induction of resolution of inflammation. In particular, the membrane bound form of CD83 is absolutely essential for the development of CD4^+^ T cells and inhibits autoimmunity *via* the induction of regulatory mechanisms which dampen ongoing or overshooting immune responses. On the other hand, the soluble CD83 protein has a great therapeutic potential to prevent/cure autoimmune disorders and to inhibit transplant rejection, *via* the induction of regulatory mechanisms, including Tregs and tolerogenic DCs. Thus, future preclinical and hopefully subsequent clinical studies will unravel the entire immune regulatory repertoire of CD83 in even greater detail and further develop the therapeutic potential of the sCD83 molecule.

## Author Contributions

LG, AW, DR, CK, EZ, IK, YM, HS, AS, and ML wrote the original draft. AS and ML edited the manuscript. YM, HS, LG, DR, and CK prepared the figures. AS and ML carried out supervision and conceptualization.

## Conflict of Interest

The authors declare that the research was conducted in the absence of any commercial or financial relationships that could be construed as a potential conflict of interest.

## Abbreviations

APC: antigen presenting cell
CD83L: CD83 ligand
DC: dendritic cell
hCD83: human CD83
Ig: immunoglobulin
IRF: interferon regulatory factor
mb: membrane-bound
mbCD83: membrane-bound form of CD83
MHC: major histocompatibility complex
Mr: relative molecular mass
muCD83: murine CD83
sCD83: soluble CD83
TFBSs: transcription factor binding sites
Tregs: regulatory T cells
URE: upstream regulatory element
UT: untranslated
wt: wild type.
